# Polygonogram with isobolographic synergy for three-drug combinations of phenobarbital with second-generation antiepileptic drugs in the tonic–clonic seizure model in mice

**DOI:** 10.1007/s43440-020-00164-5

**Published:** 2020-10-06

**Authors:** Jarogniew J. Łuszczki, Dominika Podgórska, Justyna Kozińska, Marek Jankiewicz, Zbigniew Plewa, Mateusz Kominek, Dorota Żółkowska, Magdalena Florek-Łuszczki

**Affiliations:** 1 Department of Pathophysiology, Medical University, Jaczewskiego 8b, 20-090 Lublin, PL Poland; 2 Isobolographic Analysis Laboratory, Institute of Rural Health, Lublin, Poland; 3 Chair and Clinic of Hematooncology and Bone Marrow Transplantation, Medical University, Lublin, Poland; 4 Chair and Clinic of Cardiology, Medical University, Lublin, Poland; 5 Department of General, Oncological and Minimally Invasive Surgery, 1st Military Clinical Hospital, Lublin, Poland; 6 Clinic of Orthopedics and Traumatology, Medical University, Lublin, Poland; 7 Department of Neurology, School of Medicine, University of California-Davis, Sacramento, CA USA; 8 Department of Medical Anthropology, Institute of Rural Health, Lublin, Poland

**Keywords:** Antiepileptic drugs, Drug interactions, Drug synergism, Isobolographic analysis, Maximal electroshock

## Abstract

**Background:**

Combination therapy consisting of two or more antiepileptic drugs (AEDs) is usually prescribed for patients with refractory epilepsy. The drug–drug interactions, which may occur among currently available AEDs, are the principal criterion taken by physicians when prescribing the AED combination to the patients. Unfortunately, the number of possible three-drug combinations tremendously increases along with the clinical approval of novel AEDs.

**Aim:**

To isobolographically characterize three-drug interactions of phenobarbital (PB) with lamotrigine (LTG), oxcarbazepine (OXC), pregabalin (PGB) and topiramate (TPM), the maximal electroshock-induced (MES) seizure model was used in male albino Swiss mice.

**Materials and method:**

The MES-induced seizures in mice were generated by alternating current delivered via auricular electrodes. To classify interactions for 6 various three-drug combinations of AEDs (i.e., PB + TPM + PGB, PB + OXC + TPM, PB + LTG + TPM, PB + OXC + PGB, PB + LTG + PGB and PB + LTG + OXC), the type I isobolographic analysis was used. Total brain concentrations of PB were measured by fluorescent polarization immunoassay technique.

**Results:**

The three-drug mixtures of PB + TPM + PGB, PB + OXC + TPM, PB + LTG + TPM, PB + OXC + PGB, PB + LTG + PGB and PB + LTG + OXC protected the male albino Swiss mice from MES-induced seizures. All the observed interactions in this seizure model were supra-additive (synergistic) (*p* < 0.001), except for the combination of PB + LTG + OXC, which was additive. It was unable to show the impact of the studied second-generation AEDs on total brain content of PB in mice.

**Conclusions:**

The synergistic interactions among PB and LTG, OXC, PGB and TPM in the mouse MES model are worthy of being transferred to clinical trials, especially for the patients with drug resistant epilepsy, who would benefit these treatment options.

## Introduction

Despite introduction of new therapies for treatment of seizures up to one-third of patients with epilepsy suffers from drug resistant epilepsy (medically refractory/intractable or pharmacoresistant epilepsy) [1]. In 2010 the task force of the ILAE Commission on Therapeutic Strategies proposed that drug resistant epilepsy “may be defined as failure of adequate trials of two tolerated and appropriately chosen and used AED schedules (whether as monotherapies or in combination) to achieve sustained seizure freedom” [2]. Presently, there are no evidence-based criteria or official guidance on how to choose different antiepileptic drug (AED) combinations to obtain the best therapeutic response [3].

Polytherapy with two or three AEDs is usually prescribed to epileptic patients, if their seizures are not properly controlled with one of the currently available AEDs [4–6]. Physicians are advised to combine AEDs with diverse molecular mechanisms of action based primarily on their clinical experience and information from evidence-based clinical trials [7, 8]. Unfortunately, clinical evidence provides only few combinations of AEDs that were found to be truly effective in epilepsy patients [5, 9–12]. On the other hand, clinical diversity of seizure attacks, different responses to the treatment of some specific seizures, diversity in seizure types prompt clinicians and researchers to search for novel AED combinations that would be efficacious in patients with pharmacoresistant seizures [13, 14].

Each combination of AEDs produces a pharmacodynamic interaction that can be classified as synergistic, additive, neutral or antagonistic [15, 16]. A proper classification of interactions among AEDs is usually performed by means of isobolographic analysis that is thought to be a gold standard during evaluation of pharmacodynamic interactions in preclinical experimental models of epilepsy [17]. The most favorable combinations of AEDs are those offering synergistic interactions with respect to the anticonvulsant effects [13, 18]. In contrast, the combinations of AEDs that exert antagonistic interactions in terms of the anticonvulsant effects should be avoided [13].

Of note, some of the three-drug combinations of AEDs (i.e., producing supra-additive (synergistic) interaction in experimental animals) can be used to formulate hypothesis that these combinations would be also effective in epileptic patients with seizures that are refractory to the standard AED treatment (Table 1). Undoubtedly, the preclinical studies utilizing animal seizure models may help physicians finding the most appropriate combinations of AEDs that would offer synergistic effects in suppression of seizures, if these combinations will be evaluated and their efficacy will be confirmed in further clinical trials [11, 17]. In addition, results of animal studies may point out the possible danger of some three-drug combinations of AEDs that exert infra-additive (antagonistic) interactions (Table 1).

**Table 1:** Types of interactions for the studied three-drug combinations of antiepileptic drugs in the maximal electroshock-induced seizure test in mice

Combination of three antiepileptic drugs	Type of interaction	References
Lacosamide + lamotrigine + valproate	Sub-additive	[19]
Lacosamide + carbamazepine + valproate	Sub-additive	[20]
Lacosamide + carbamazepine + lamotrigine	Additive	[21]
Lacosamide + carbamazepine + phenobarbital	Additive	[22]
Lacosamide + lamotrigine + phenobarbital	Additive	[23]
Carbamazepine + phenobarbital + valproate	Additive	[24]
Carbamazepine + phenobarbital + topiramate	Supra-additive	[25]
Oxcarbazepine + pregabalin + topiramate	Supra-additive	[26]
Phenobarbital + phenytoin + pregabalin	Supra-additive	[27]

In this study, we utilized isobolographic experiments to evaluate the anticonvulsant effectiveness of three-drug combinations of currently available AEDs in preclinical seizure model. The main purpose of this study was to evaluate the types of interactions for 6 various three-drug combinations, in which phenobarbital (PB—a classical AED) was a leading drug. Previously, we have reported that some three-drug combinations containing PB, exerted both additive and supra-additive interactions in experimental animals (Table 1). To determine the interaction profiles for three-drug combinations of AEDs, we tested PB in combination with various second-generation AEDs including, lamotrigine (LTG), oxcarbazepine (OXC), pregabalin (PGB) and topiramate (TPM). The effectiveness of 6 selected AED combinations (i.e., PB + TPM + PGB, PB + OXC + TPM, PB + LTG + TPM, PB + OXC + PGB, PB + LTG + PGB and PB + LTG + OXC) with respect to seizure suppression was assessed in the mouse maximal electroshock-induced seizure (MES) model by means of the type I isobolographic analysis of interaction, as described elsewhere [19, 21, 22, 25–27]. The choice of PB and the second-generation AEDs (LTG, OXC, PGB and TPM) to determine the interaction profiles of all possible three-drug combinations (i.e., PB + TPM + PGB, PB + OXC + TPM, PB + LTG + TPM, PB + OXC + PGB, PB + LTG + PGB, and PB + LTG + OXC) was based primarily on various molecular mechanisms of action of these AEDs [28, 29], and their impact on suppression of both, experimentally MES-induced seizures in animals and generalized (tonic–clonic) seizures in epilepsy patients [30, 31].

## Materials and methods

### Animals and drug administration

All procedures involving animals complied with the ARRIVE guidelines and were approved by the Local Ethics Committee (Lublin, Poland). Totally, 424 adult male albino Swiss outbred mice (weighing 20–26 g) were used in this study. More specifically, 328 mice were studied in the MES test and 96 mice were tested during the measurement of AED concentrations. LTG (Glaxo Wellcome, Kent, UK), OXC (Novartis Pharma AG, Basel, Switzerland), PB (Polfa, Krakow, Poland), PGB (Pfizer Ltd., Sandwich, Kent, UK) and TPM (Cilag AG, Schaffhausen, Switzerland) were suspended in a 1% aqueous solution of Tween 80 (Sigma-Aldrich, Poznan, Poland). All the AEDs were administered systemically (ip*)* in a volume of 5 ml/kg body weight. OXC was injected 30 min., LTG, PB and TPM–60 min. and PGB–120 min. prior to the MES test, as recommended elsewhere [32–34].

### Maximal electroshock-induced seizure (MES) model

Maximal electroshock-induced seizures in experimental animals were evoked by alternating current (50 Hz, 25 mA, 500 V, 0.2 s stimulus duration) using auricular electrodes. The seizure activity in experimental animals manifested in forms of tonic hind limb extension. Doses of the investigated AEDs were transformed to their logarithms to the base 10, whereas the anticonvulsant effects produced by the drugs were transformed to their respective probits, as recommended elsewhere [35]. Thus, the median effective doses (ED_50_ values ± SEM) of the investigated AEDs (that suppressed tonic seizures in 50% of the mice) were determined, as it was described earlier [35]. Similarly, by transforming increasing doses of the three-drug mixtures for the respective AED combinations (in the fixed-ratio combination of 1:1:1) to the logarithms, and the anticonvulsant activity produced by the three-drug combinations from the MES test to their respective probits, it was possible to determine the experimentally-derived median effective doses (ED_50 mix_ values ± SEM) for the studied three-drug combinations against electrically evoked seizures in the MES test, as described earlier [22, 24, 26, 36].

### Type I isobolographic analysis

Pharmacodynamic interactions for the three-drug combinations of AEDs administered ip in the fixed-ratio combination of 1:1:1 were evaluated by means of the isobolographic analysis, as described earlier [19–27]. Subsequently, the additive median effective doses (ED_50 add_ values ± SEM) for the three-drug combinations of AEDs were calculated mathematically from the general equation of additivity based on the mass-action law according to Loewe [37], as described elsewhere [38]. To calculate the SEM values for the ED_50_ values, we used equations allowing such transformation, as published elsewhere [39, 40].The experimentally-derived ED_50 mix_ values and the theoretically-calculated ED_50 add_ values were statistically compared with the unpaired Student’s *t*-test with Welch’s correction by the use of GraphPad Prism 7.0 (GraphPad Software, San Diego, CA, USA).

### Pharmacokinetic measurement of total brain concentrations of PB

Total brain content of PB was determined in mice that were administered PB alone or in combinations with other tested AEDs at the fixed-ratio of 1:1:1 from the mouse MES model. Mice were killed by decapitation at times chosen to coincide with that scheduled for the MES-induced seizure test and whole brains were removed from skulls, weighed, and homogenized using Abbott buffer (2:1 vol/weight; Abbott Laboratories, North Chicago, IL, USA). The homogenates were centrifuged at 10,000 g for 10 min, and the supernatant samples (300 μl) were analyzed by immunofluorescence technique. Total brain concentrations of PB are expressed in μg/ml of brain supernatants as means ± SEM of at least 8 separate brain preparations. The difference between the PB content in animals receiving PB alone or PB in three-drug mixture was statistically analyzed with the unpaired Student’s *t*-test.

## Results

### Anticonvulsant effects of the studied AEDs in the mouse MES model

**Fig. 1:**
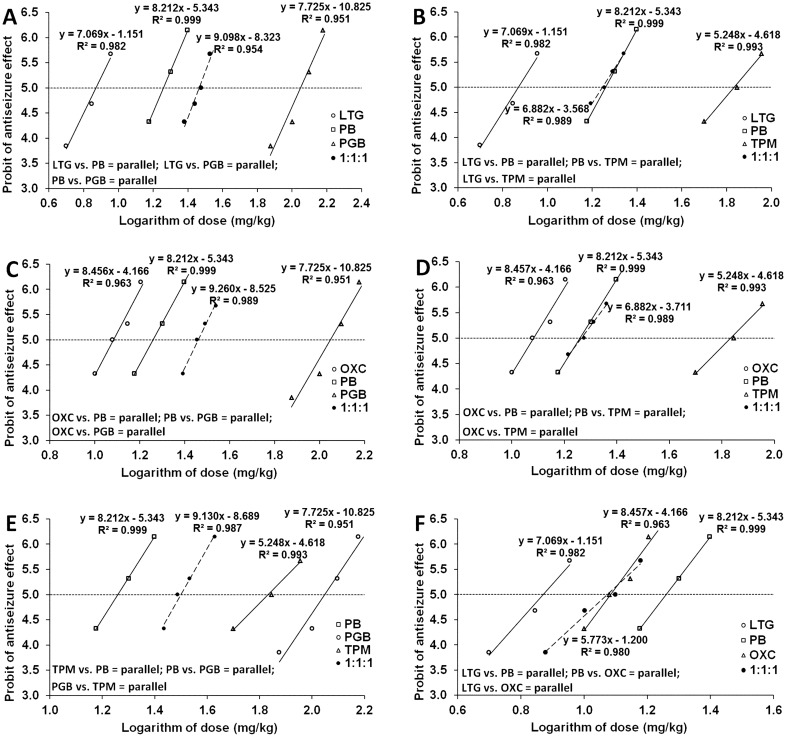
**a**–**f** Phenobarbital (PB), lamotrigine (LTG), oxcarbazepine (OXC), pregabalin (PGB), topiramate (TPM), and their combination (at the fixed-ratio of 1:1:1) in the tonic–clonic seizure (MES) model in mice. Logarithms of doses of PB, LTG, TPM, PGB, OXC, and the three-drug combinations were plotted on X-axis, while the anticonvulsant effects of the tested AEDs were plotted on Y-axis. Dose–response relationship effects were linearly related for the investigated AEDs and their combinations. The horizontally placed 5th probit illustrates median effective doses (ED_50_) of AEDs when intersecting with dose–response lines for particular drugs

All the investigated AEDs suppressed (in a dose-dependent manner) MES-induced seizures in animals and their median effective doses (ED_50_ values), when administered ip separately, were 18.17 ± 1.80 mg/kg for PB, 7.42 ± 0.85 mg/kg for LTG, 12.13 ± 0.95 mg/kg for OXC, 111.82 ± 9.61 mg/kg for PGB and 68.04 ± 8.61 mg/kg for TPM, respectively (Fig. 1a–f).

### Isobolographic analysis of interaction for three-drug combinations of AEDs

When isobolographically compared the experimentally-derived ED_50 mix_ values with their respective theoretically-calculated ED_50 add_ values at the fixed-ratio of 1:1:1, supra-additive (synergistic) interactions were documented for all the studied three-drug combinations, except for the combination of PB + LTG + OXC, which was additive (Table 2).

**Table 2:** Isobolographic analysis of interaction for 6 three-drug combinations of AEDs at the fixed-ratio of 1:1:1 in the MES-induce seizure model in mice

Combination	ED_50 mix_	*n*_mix_	ED_50 add_	*n*_add_	Unpaired *t*-test	Ω
PB + TPM + PGB	31.58 ± 2.30 ***	24	66.01 ± 3.84	58	t_79.79_ = 7.692; *p* < 0.0001	0.48
PB + LTG + TPM	17.58 ± 1.47 ***	32	31.21 ± 2.88	50	t_70.31_ = 4.215; *p* < 0.0001	0.56
PB + OXC + TPM	18.44 ± 1.54 ***	32	32.78 ± 2.88	58	t_81.94_ = 4.391; *p* < 0.0001	0.56
PB + OXC + PGB	28.88 ± 1.79 ***	32	47.37 ± 3.21	58	t_83.17_ = 5.031; *p* < 0.0001	0.61
PB + LTG + PGB	29.13 ± 1.84 ***	32	45.80 ± 3.21	50	t_73.88_ = 4.505; *p* < 0.0001	0.64
PB + LTG + OXC	11.86 ± 1.36	24	12.57 ± 0.65	50	t_33.88_ = 0.471; *p* = 0.6406	0.94

Values are median effective doses (ED_50_ ± SEM) of three-drug mixtures, administered at the fixed-ratio of 1:1:1, in the MES-induced seizure model in mice. The ED_50 mix_ values (in mg/kg) are experimentally-derived doses of the three-drug combinations that protected 50% of the tested animals from tonic seizures, while the ED_50 add_ values (in mg/kg) are doses of the three-drug combinations expected to produce additive interaction, when calculated from a theoretical equation of additivity. Both, n_mix_ and n_add_ values indicate number of animals at those doses, whose anticonvulsant effects ranged from 4 to 6 probits. Ω–interaction index is a ratio of ED_50 mix_ and ED_50 add_ values. ***p < 0.001 vs the respective ED_50 add_ value (with unpaired Student’s *t*-test).

**Fig. 2:**
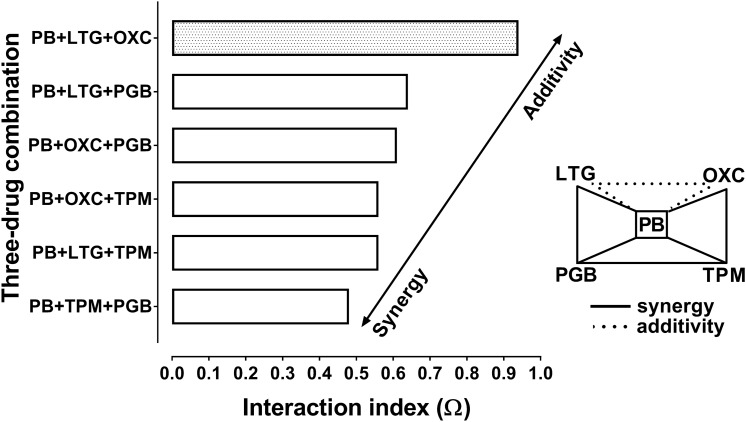
Polygonogram for three-drug combinations illustrating synergistic and additive interactions among selected antiepileptic drugs in the tonic–clonic seizure (MES) model in mice. Polygonogram for 5 AEDs with different mechanisms of actions. Phenobarbital (PB), lamotrigine (LTG), pregabalin (PGB), topiramate (TPM), oxcarbazepine (OXC), were combined together and the three-drug mixtures (at the fixed-ratio of 1:1:1) underwent isobolographic evaluation in the mouse MES model. Horizontal columns represent interaction index values for the studied three-drug combinations. Solid line indicates synergism among the investigated AEDs, whereas the dotted line illustrates additive interaction

The Student’s *t*-test with Welch’s correction revealed that synergy was observed at p < 0.001 for all the investigated combinations of AEDs, except for the combination of PB + LTG + OXC (Table 2). Additionally, the interaction index (Ω–calculated as a ratio of the respective ED_50 mix_ and ED_50 add_ values) ranged from 0.48 to 0.94, indicating synergistic and additive interactions among the studied AEDs (Fig. 2).

### Brain AED concentrations

Total brain concentrations of PB administered alone did not differ significantly from those determined for the combinations of PB + TPM + PGB, PB + LTG + TPM, PB + OXC + TPM, PB + OXC + PGB, PB + LTG + PGB and PB + LTG + OXC from the MES model (Table 3).

**Table 3:** Influence of combinations of AEDs on total brain concentrations of PB in experimental mice

Treatment (mg/kg)	Total brain concentrations (μg/ml)		*n*	Unpaired *t*-test
PB (2.90) + vehicle + vehicle	1.23 ± 0.37		8	
PB (2.90) + TPM (10.85) + PGB (17.83)	1.40 ± 0.45	↑ 14%	8	t_14_ = 0.292; *p* = 0.775
PB (3.41) + vehicle + vehicle	1.54 ± 0.47		8	
PB (3.41) + OXC (2.27) + TPM (12.76)	1.71 ± 0.62	↑ 11%	8	t_14_ = 0.219; *p* = 0.830
PB (3.41) + vehicle + vehicle	1.54 ± 0.47		8	
PB (3.41) + LTG (1.39) + TPM (12.78)	1.79 ± 0.68	↑ 16%	8	t_14_ = 0.302; *p* = 0.767
PB (3.69) + vehicle + vehicle	1.69 ± 0.418		8	
PB (3.69) + OXC (2.46) + PGB (22.72)	1.91 ± 0.70	↑ 13%	8	t_14_ = 0.271; *p* = 0.790
PB (3.85) + vehicle + vehicle	1.81 ± 0.528		8	
PB (3.85) + LTG (1.57) + PGB (23.17)	2.05 ± 0.77	↑ 13%	8	t_14_ = 0.258; *p* = 0.800
PB (5.71) + vehicle + vehicle	2.88 ± 0.918		8	
PB (5.71) + LTG (2.33) + OXC (3.81)	2.97 ± 0.96	↑ 3%	8	t_14_ = 0.068; *p* = 0.947

Data are presented as means (± SEM) and expressed as µg/ml of brain supernatants

## Discussion

Current study provided experimental evidence that combinations of three AEDs containing one classical AED—PB, produced synergistic interaction in the mouse MES model. Almost all the examined combinations (i.e., PB + TPM + PGB, PB + OXC + TPM, PB + LTG + TPM, PB + OXC + PGB and PB + LTG + PGB) exerted synergistic interactions in preclinical studies. Only one combination of PB + LTG + OXC was additive. Considering molecular mechanisms of action of the investigated drugs one can observe that PB-mediated activation of GABA_A_ receptors contributed to the synergistic suppression of seizures in animals. In addition, diverse molecular mechanism of action of the second-generation AEDs (OXC, TPM, LTG and PGB) undoubtfully contributed to observed synergy. A basic question arises whether we really need combinations of 3 AEDs for patients with epilepsy. While the refractory epilepsy is still the challenging issue for clinicians and physicians nowadays, increasing number of AEDs (available and licensed as effective treatment options for epilepsy patients) offers tremendous number of possible two or three AEDs combinations. Do we really need combinations of 3 AEDs to stop seizure initiation and propagation in the human brain? As drug resistant epilepsy remains an extraordinary therapeutic challenge the answer to above question may only be positive. With more than 25 AEDs that are currently available for epilepsy treatment the number of possible combinations remains very high. Selection of AEDs for combined treatment should fulfill the basic concepts associated with diverse molecular mechanisms of action of chosen AEDs [41]. At present, it is recommended that AEDs should be combined together if their molecular mechanisms of action are quite different [42]. Activation of several mechanisms of the anticonvulsant action within combined AEDs significantly increases the probability of successful seizure suppression in treated patients [13]. Thus, there is still urgent need of AEDs that present various molecular mechanisms of action in terms of suppression of seizures. General recommendation from preclinical in vivo studies conducted on animals by means of the isobolographic analysis indicated that the synergistic interaction occurs among three AEDs, if at least one of the drug possesses molecular mechanisms of action related with a direct activation of GABA-ergic system and GABA_A_-receptor-mediated response [28]. Thus, the PB-induced suppression of seizures can readily accompany the effects of drugs whose main mechanisms are linked to the blockade of sodium channels (OXC, LTG) in neurons and inhibition of seizure activity propagation [28]. Recommendations may include drugs that also block calcium channels in neurons and thus, inhibit presynaptic liberation of excitatory neurotransmitters in neurons (PGB) [29]. Besides, favorable combinations of AEDs were reported for the drugs exerting several molecular mechanisms of action (i.e., with multi-targets of their anticonvulsant activity—TPM) [43–45]. From a theoretical point of view, the most recommended combinations should consist of PB or one of the GABA_A_-receptor activating drugs, LTG or OXC – or one of the sodium channel blockers, which inhibits seizure propagation in the brain and one of the multi-targets drugs like TPM, with various molecular mechanisms of action. Additionally, instead of sodium channel blockers, sometimes calcium channel blockers may also occur favorable, like PGB. The theoretically-selected combinations of 3 AEDs were verified isobolographically whether the observed interactions can be classified as favorable (synergistic) in the mouse MES model. In case of the additive interaction observed for the combination of PB + LTG + OXC, one can ascertain that LTG and OXC have quite similar molecular mechanisms of action associated with blockade of sodium channels in neurons [46, 47]. Thus, both AEDs compete one another with target sites within the sodium channels and both drugs (OXC and LTG) were not able to exceed the effect exerted by PB in terms of suppression of seizures and thus, the final effects for the combination of PB + LTG + OXC was only additive in the MES test in mice.

Experiments conducted on animals confirmed our theoretical expectations providing a proof of efficacy of these AEDs in combinations. Selection of PB (the classic AED) was based primarily on its molecular mechanisms of action connected to GABA_A_-receptor-mediated inhibition of seizure activity. The choice of second-generation AEDs (including, LTG, OXC, PGB and TPM) was mainly based on their various molecular mechanisms of action and their favorable safety profiles with no acute adverse effects accompanying the therapy with classical AEDs.

A general rule for the AEDs combinations indicates that the less adverse effects are, the more tolerable combination is for the patients. Development of tolerance to the anticonvulsant properties of AEDs when used in combination is still a challenging issue for physicians nowadays. They need some stable combinations, whose anticonvulsant effect does not change during the time of the therapy. The stability of treatment with AEDs that persists in time seems to be the most desirable outcomes for the patients with epilepsy.

In this study, we calculated the interaction index, which is an indicator of the strength of interaction among 3 AEDs. Generally, the interaction index value lower than 0.7 indicates synergistic interaction among the AEDs [48, 49]. The lower interaction index value is, the stronger synergy is documented among the AEDs in preclinical studies [49, 50]. In this study, all the combinations of AEDs had their interaction indices lower than 0.7, except for the combination of PB + LTG + OXC, for which the interaction index amounted to 0.94 (Table 2). Additionally, we displayed, for the first time, the interaction occurring among 3 AEDs by means of polygonogram (Fig. 2), which is thought to be a simple graphical visualization of interactions occurring among drugs [50, 51]. Polygonogram illustrates interactions among the investigated drugs tested in one fixed-ratio combination of 1:1:1, where doses of particular AEDs produced equal effects in terms of seizure suppression in the mouse MES model. Additionally, in our study, PB was a leading drug present in all combinations and therefore, it was placed in the center of the polygonogram (Fig. 2).

Previously, it has been documented that some three-drug combinations containing PB (i.e., PB + PHT + PGB and PB + CBZ + TPM) were also synergistic in the mouse MES model (Table 1). Two other three-drug combinations with PB (i.e., LCM + CBZ + PB and CBZ + PB + VPA) have been reported to be additive in the mouse MES model (Table 1). Unfortunately, the evaluation of interaction in clinical conditions is not possible because of numbers of combinations of AEDs theoretically available. Presently, physicians can treat epilepsy by means of 25 various AEDs [52]. However, these 25 AEDs can generate 300 various two-drug combinations and 2,300 three-drug combinations. So, it is unlikely to verify them all in clinical trials.

To provide a more systematic approach to the problem of three-drug combinations, it is worthy of mentioning the isobolographic types of interactions occurring between two AEDs in the same experimental model of epilepsy. Characteristics of interactions between two AEDs in the MES test revealed that 3 out of 10 two-drug combinations were synergistic in mice (i.e., PB + LTG, TPM + OXC and TPM + LTG—Table 4). Unfortunately, one of the studied two-drug combinations occurred antagonistic in the mouse MES model (i.e., OXC + LTG—Table 4). The other two-drug combinations remained additive in animals subjected to the mouse MES model (Table 4).

**Table 4:** Isobolographic characteristics of two-drug interactions (at the fixed-ratio of 1:1) among the studied AEDs in the mouse tonic–clonic seizure model

Drug combination	Type of interaction	Reference
PB + LTG	Synergy	[53]
PB + OXC	Additivity	[40]
PB + PGB	Additivity	[54]
PB + TPM	Not tested	–
OXC + LTG	Antagonism	[16]
OXC + PGB	Additivity	[55]
PGB + LTG	Additivity	[55]
TPM + LTG	Synergy	[53]
TPM + OXC	Synergy	[16]
TPM + PGB	Additivity	[55]

Bearing in mind the antagonistic interaction occurring between OXC and LTG in the mouse MES model [16], one can readily explain the additivity observed for the combination of PB with OXC and LTG. It seems that PB-mediated suppression of tonic–clonic seizures in mice was strong enough to replace the antagonistic interaction produced by OXC + LTG with additivity in the MES test.

It should be stressed that in this study we measured only total brain concentrations of PB—the classical AED present in all three-drug mixtures. With fluorescent polarization immunoassay method, we were unable to detect any significant difference in PB concentrations between animals receiving PB alone or PB with two-drug combinations (i.e., TPM + PGB, OXC + TPM, LTG + TPM, OXC + PGB, LTG + PGB and LTG + OXC). Moreover, doses of the AEDs that exerted synergistic interaction in the mouse MES model were low enough to be able to exert any pharmacokinetic interactions with other second-generation AEDs co-administered. To entirely exclude pharmacokinetic interactions among AEDs, the content of all co-administered AEDs should be evaluated in the mouse brain tissue. Since we did not measure total brain concentrations of the second-generation AEDs, we cannot entirely exclude the existence of pharmacokinetic interactions occurring among AEDs. It is unlikely that PGB or TPM were able to pharmacokinetically interacted with other AEDs, especially with OXC or LTG, when combined and vice versa. Accumulating pharmacokinetic studies indicate that PGB does not undergo metabolic transformation and is eliminated as unchanged drug in the urine [56]. LTG is metabolized in the liver by UDP-glucuronosyltransferase [57]. OXC as a pro-drug is metabolized to monohydroxy derivative (MHD) of carbamazepine [58]. TPM is metabolized in the liver by CYP isoenzymes [59]. Since pharmacokinetic profiles of the second-generation AEDs differ considerably, it is unlikely that AEDs would be able to pharmacokinetically interact with other co-administered AEDs. Additionally, it is unlikely that three-drug mixtures comprising PB and various constellation of second-generation AEDs (LTG, OXC, PGB and TPM) would be able to pharmacokinetically interact and mutually exert pharmacokinetic interactions among the AEDs. Of note, the measurement of PB concentrations in the brain tissue was performed when AEDs were administered acutely (as single injections). We are fully aware of the fact that during chronic administration of the AEDs some pharmacokinetic interactions occur. This is the reason that both, isobolographic analysis of interactions in the mouse MES model and measurement of total brain concentrations were performed in this study, when AEDs were administered systemically (ip) as acute injections.

Another fact needs additional explanation while investigating any potential pharmacokinetic changes in PB content in the mouse brain tissue. The same formulation of PB (i.e., 1% solution of Tween 80 in distilled water), and the same pretreatment time after ip injection of PB (i.e., 60 min) either alone or in combination with AEDs, were used in this study. Of note, the 3 AEDs when combined were never mixed in one syringe before injection, but the AEDs were administered as 3 separate injections (each drug was injected singly and separately) to exclude any pharmaceutical interactions that could occur among the studied AEDs when mixed together [41, 60]. Additionally, total brain concentrations of PB were measured in drug-naïve animals to exclude any prior inhibition or activation of liver enzymes involved in metabolism of AEDs that could potentially change pharmacokinetic profile of PB. In this study we observed, however, a slight, but insignificant increase in PB content in the brains of experimental animals. Each AED combinations investigated in this study increased PB level that ranged between 3% up to 16% (Table 3). Such increases may hypothetically result from a slight (non-significant) inhibition of liver enzymes (CYP450) after co-administration of other AEDs. However, to confirm this hypothesis, more advanced pharmacokinetic studies are needed. On the other hand, by definition in bioequivalence analysis, similarity of concentrations of a drug is reported if the measured drug concentrations with 90% confidence intervals lie in the range 80–125% [61]. In our study, the maximal increase in total brain PB concentrations amounted to 16% and it can be placed within the limited range, confirming similarities between the PB concentrations. Besides, the same preparations of PB was used when the drug was injected alone or when it was injected in combination with other AEDs. Thus, any difference in bioavailability of PB can be excluded, as a main cause of slight increase in PB content in the mouse brain tissue.

The main limitation in this study is the acute model of seizures electrically evoked in experimental drug-naïve animals. Of note, some models of epilepsy using phenytoin- or LTG-resistant kindled rats, 6 Hz corneal stimulation induced seizures in mice or post status epilepticus spontaneous recurrent seizures [62], displaying varying degrees of pharmacoresistance would be suitable to test response of 3 AEDs in mixtures, similarly, as it is observed in clinical circumstances. Also the AEDs should be administered chronically to mimic all clinical conditions and circumstances and exclude any kind of untoward side effects, pharmacokinetic interaction, etc. But, this study was purportedly designed to screen whether or not three-drug combinations are synergistic when the drugs are combined together. Generally, when using a screening test for selecting novel potentially active compounds with anticonvulsant properties, researchers use the MES model as a first screen test for selecting active candidates that undergo other experimental verification with tests mimicking pharmacoresistance [63–65]. This was the main reason to investigate triple AED combinations in the mouse MES model so as to choose only favorable combinations exerting only synergistic interactions with respect to the anticonvulsant protection from seizures.

Another limitation in this study is clinical application of PB that is currently used in pediatric population and rarely in adult patients [66, 67]. Although its clinical application to humans is limited, in this study we chose PB because of its pharmacologic properties affecting directly GABA-ergic neurotransmission in the brain. PB can be considered a model drug testing its GABA_A_-mediated anticonvulsant response with other second-generation AEDs.

Clinicians are also aware of the fact that addition of the third AED to the duotherapy provides a chance for only 5–10% improvement [10–12], therefore, the triple therapy in epilepsy is seldom prescribed. However, from the refractory patient’s perspective any improvement is considered as a great success and this is the reason that novel drug candidates, novel AED combinations are investigated to improve the patient’s life. Last, but not least limitation in this study is preclinical condition verified in animals. It would be better to verify triple interactions directly on humans in clinical settings, but difficulties with enrollment of the patients with the same seizure types and similar history of disease to experimental groups could disturb or even make impossible such investigations.

## Conclusions

Summing up, the three-drug combinations of PB + TPM + PGB, PB + OXC + TPM, PB + LTG + TPM, PB + OXC + PGB and PB + LTG + PGB, due to synergistic interactions observed in the mouse MES model, may be worthy of consideration by clinicians while treating patients with drug resistant epilepsy. The combination of PB + LTG + OXC produce additive interaction. Isobolographic verification of the exact types of interaction among three AEDs in combination should be required to confirm beneficial effects related with polytherapy with AEDs.

## Data Availability

All data will be available upon request.
